# Trigeminal postherpetic neuralgia responsive to treatment with capsaicin 8 % topical patch: a case report

**DOI:** 10.1007/s10194-012-0467-0

**Published:** 2012-06-21

**Authors:** Jennifer Sayanlar, Nilufer Guleyupoglu, Russell Portenoy, Sait Ashina

**Affiliations:** 1 Department of Pain Medicine and Palliative Care, Albert Einstein College of Medicine, Beth Israel Medical Center, New York, NY USA; 2 Department of Neurology, Albert Einstein College of Medicine, Beth Israel Medical Center, New York, USA

## Abstract

Postherpetic neuralgia has been variably defined but is generally understood to be pain that persists for longer than a few months after an attack of herpes zoster. Pain persists for years in approximately 10 % of those afflicted with acute herpes zoster. The likelihood of postherpetic neuralgia increases with older age, severity of the zoster, trigeminal location, and other factors. Postherpetic neuralgia is a neuropathic pain and treatment usually involves sequential trials of topical and systemic drugs; a variety of other therapies may be considered in refractory cases. A new topical capsaicin 8 % patch has been approved for this indication based on the positive studies in patients with non-trigeminal postherpetic neuralgia. Experience with the use of the capsaicin 8 % patch for trigeminal distribution neuralgia is lacking. We report a case of trigeminal postherpetic neuralgia which was safely and effectively treated with capsaicin 8 % patch.

## Introduction

Postherpetic neuralgia (PHN) is the persistence of pain after the disappearance of the rash caused by the herpes zoster virus [1]. The definition of PHN has been controversial, ranging from pain persisting after the healing of rash to pain that continues beyond a period that varies between 1 and 6 months [2]. Risk factors for PHN include older age, immunosuppression, female gender, presence of prodrome, greater rash severity, and greater acute pain [3–5]. The thoracic dermatomes are most commonly affected, followed by the region innervated by the ophthalmic division of the trigeminal nerve [6, 7], particularly that supplied by the frontal branch of V1 [1].

Although PHN may decline over time, chronic pain continues indefinitely in approximately 10 % of those afflicted. For many patients, the chronic neuropathic pain has a profound adverse effect on function and quality of life. Numerous treatments are considered, including both topical therapies and systemic treatment with a variety of drugs. Antidepressants, gabapentin, pregabalin, opioids, and topical lidocaine have been shown to be efficacious in randomized controlled studies [8, 9] and many other drug therapies, interventional approaches, and psychological or rehabilitative treatments are tried empirically in those with persistent pain [10–13].

Low concentration topical capsaicin has been used for decades as a treatment for PHN, with equivocal results [14]. In recent years, a high concentration (8 %) capsaicin patch has been developed, and approved for PHN based on two randomized controlled studies demonstrating the efficacy and safety of a 1-h application for PHN in non-trigeminal dermatomal distributions [15, 16]. This treatment may reduce pain for many months and the controlled trials suggest that a typical responder will experience improvement for approximately 3 months.

The capsaicin 8 % patch has not been studied for trigeminal PHN, and the feasibility, safety and efficacy of the patch when used on the face and head remains to be determined. We present a case of PHN in the trigeminal nerve dermatomal distribution which responded to treatment with capsaicin 8 % topical patch.

## Case

A 64-year-old woman with past medical history of depression, migraine, gastroesophageal reflux disease, carpal tunnel syndrome and benign breast mass removal initially presented to the Department of Pain Medicine and Palliative Care at Beth Israel Medical Center with right facial pain following a V1 trigeminal herpes zoster eruption approximately 4 weeks earlier. The acute infection was complicated by an anterior uveitis and altered mental status associated with an abnormal MRI, specifically high T2 signal in bilateral superficial occipital lobes. Although cerebrospinal fluid was normal, the patient was treated with high dose IV acyclovir for 10 days for a suspected herpes zoster encephalitis. She rapidly improved, clinically and radiographically, and was switched to oral valacyclovir for a brief additional period.

Pain persisted after the acute infection resolved. Trials of an opioid, pregabalin, and a combination product containing butalbital, acetaminophen, and caffeine were ineffective. At the time of presentation to the pain practice, the patient complained of continuous severe pain in the right upper cheek, eye, and forehead, with radiation to the right temporal region. The pain was described as a constant burning with intermittent electrical shock-like painful sensations occurring approximately every 10 min, each lasting several seconds. The burning pain was rated as an 8/10 on an 11-point verbal numerical pain rating scale. The affected area was sensitive to touch, heat (e.g., when washing hair with hot water), and combing hair. The patient had difficulty with opening her right eye. The pain greatly interfered with her ability to function and she was very dysphoric.

On the initial examination, the skin was discolored and erythematous in the right V1 distribution. The affected area demonstrated hyperalgesia and allodynia. Otherwise, the neurological examination was normal.

The patient was diagnosed with trigeminal PHN based on the history of persistent pain (lasting more than 1 month since the acute infection resolved). Pregabalin was continued (highest dose of 150 mg orally three times daily) and additional drugs were tried, including duloxetine (highest dose of 60 mg daily), tapentadol (highest dose 200 mg daily), oxymorphone (highest dose 20 mg daily), and morphine sulfate extended release (highest dose of 60 mg daily). With the exception of morphine, which provided slight benefit, the latter drugs did not provide more relief at the maximum tolerated doses. The patient was also briefly treated with gabapentin but had to discontinue the medication due to adverse effects.

Approximately 6 weeks after the zoster eruption, pain was still severe despite treatment with pregabalin and morphine. The patient consented to a trial of topical capsaicin treatment. She received a single 60-min application of the topical capsaicin 8 % patch (Qutenza^®^) applied to the right side of her forehead, sparing the area around the eye. The total dose of capsaicin in the patch was 179 mg. Safety goggles were worn during the application to further protect the eyes. The skin to which the patch was applied was pretreated with 20 g of topical anesthetic lidocaine/prilocaine 2.5 %/2.5 %. Aside from transient erythema and tolerable application-related pain, the patient tolerated the procedure well.

Within a few days, she reported substantial pain relief. At a 1-month follow-up, she indicated that the pain could be graded at a score of 4–5/10 and the area of pain was reduced in size, involving a much smaller area on the forehead. Allodynia also was improved, with only some small residual patchy allodynic regions left. The patient was able to open her right eye more with improved visual acuity and was able to wash and comb her hair.

Approximately 1 year after the procedure the patient reported the pain score was 1/10, and her medications included pregabalin 75 mg orally three times daily, duloxetine 60 mg daily and morphine sulfate extended release total dose of 30 mg daily. Duloxetine was also used for the treatment of depression. She complained of persistent itch and the sensation of a “piece of sand” in the right eye at 1-year follow-up. She continues to be followed by the pain practice and is undergoing tapering of her analgesics.

## Discussion

The topical capsaicin 8 % patch provided prompt and meaningful relief for this patient with trigeminal PHN. Systemic treatment with pregabalin and an opioid yielded insufficient relief until the treatment with topical capsaicin, after which the patient experienced substantial improvement without initial change in her drug regimen. After a period of reduced pain, the medications could be tapered without clinical worsening. The pain relief was satisfactory and associated with improved functioning.

The long-lasting relief of pain in this case was surprising, but the potential for this outcome was suggested by a previous 1-year extension study [17]. The latter study showed that the cohort of patients given up to four capsaicin 8 % topical patch applications experienced a prolonged period of reduced pain. It was found that a decrease in pain scores from baseline was maintained with subsequent capsaicin 8 % similar topical patch treatments regardless of the number of treatments received [17].

There is no published experience with the capsaicin 8 % patch for trigeminal distribution pain. The feasibility and safety of the approach depends on the ability to fit the patch to the contours of the affected area while avoiding contact with the eyes. This case suggests that the treatment may be undertaken, with only transient side effects similar to those previously reported for non-trigeminal cases [15–17].

Capsaicin (trans-8-methyl-*N*-vanillyl-6-nonenamide) is the principal substance in hot chili peppers and has been in clinical use as a cream to treat various types of painful conditions [18, 19]. Capsaicin is a highly selective and potent agonist for the transient receptor potential vanilloid 1 (TRPV1) receptor, a trans-membrane receptor-ion channel complex, which is involved in responses to nociceptive stimuli and is gated by noxious heat temperature, low pH, and endogenous lipids [19, 20]. The exact mechanism of action of topical capsaicin in pain relief is not fully understood. It has long been thought that the pain effects may be due to the reduction of substance P content in the skin [21], but the recent studies demonstrate that topical capsaicin also causes defunctionalization of upregulated and sensitized TRPV1 receptors on sensory nerve endings [22–24].

This case suggests that the topical capsaicin 8 % patch can be used in the treatment of trigeminal PHN. With efforts to protect the eyes and fully apply the patch to the affected region, the clinical utility of the patch for PHN in this distribution should parallel that demonstrated in non-trigeminal conditions. Further study will be needed to confirm this.
